# Cloud Point Behavior of Poly(trifluoroethyl methacrylate) in Supercritical CO_2_–Toluene Mixtures

**DOI:** 10.3390/molecules30061199

**Published:** 2025-03-07

**Authors:** James R. Zelaya, Gary C. Tepper

**Affiliations:** Department of Mechanical and Nuclear Engineering, Virginia Commonwealth University (VCU), Richmond, VA 23284, USA; zelayacanajr@vcu.edu

**Keywords:** scCO_2_, poly(trifluoroethyl methacrylate) (poly(TFEMA)), cosolvent, toluene, cloud point, phase behavior, polymer solubility, Tait equation, supercritical fluid, fluoropolymer

## Abstract

Supercritical CO_2_ (scCO_2_) is a versatile solvent for polymer processing; however, many partially fluorinated polymers exhibit limited solubility in neat scCO_2_. Organic cosolvents such as toluene can enhance polymer–solvent interactions, thereby improving solubility. The cloud point behavior of poly(2,2,2-trifluoroethyl methacrylate) (poly(TFEMA)) at 3 wt% concentration in scCO_2_–toluene binary mixtures was investigated over a temperature range of 31.5–50 °C and toluene contents of 0–20 wt%. Solvent mixture densities were estimated using the Altuin–Gadetskii–Haar–Gallagher–Kell (AG–HGK) equation of state for CO_2_ and the Tait equation for toluene. For all compositions, the cloud point pressure was observed to increase linearly with temperature. The cloud point pressure decreased monotonically with increasing toluene concentration and at the highest concentration of 20 wt% was reduced by approximately 40% in comparison to neat scCO_2_. The addition of toluene lowered the solvent density, but the increase in solvent–solute molecular interactions resulted in the observed decrease in cloud point pressure. Toluene is shown to be an effective cosolvent for dissolving poly(TFEMA) in scCO_2_, offering a promising approach to lowering operating pressures in fluoropolymer processing. Our results provide valuable phase behavior data for designing scCO_2_-based extraction, impregnation, and particle formation processes involving poly(TFEMA).

## 1. Introduction

scCO_2_ is widely recognized as a versatile and environmentally benign solvent due to its tunable density, gas-like diffusivity, and liquid-like solvating power [1,2,3]. Although CO_2_ is a greenhouse gas, its use as a solvent in polymer processing is carbon neutral, since no additional carbon dioxide is produced. Consequently, it is considered environmentally “green”. These unique properties have enabled a broad range of applications, including high-purity film deposition [4,5,6,7], nanostructured material synthesis [8,9,10], polymer impregnation [11], supercritical fluid extractions [12,13,14,15], and the exfoliation of layered materials [16,17]. Despite its many advantages, scCO_2_ often exhibits insufficient solvating capacity for high-molecular-weight or polar polymers, necessitating high operating pressures to improve dissolution [18,19,20].

A widely adopted strategy to mitigate the solvating limitations of scCO_2_ while preserving most of its advantages is the use of a cosolvent to enhance solvent–solute interactions [12,21,22,23,24,25]. Recent studies have further highlighted how organic cosolvents influence polymer solubility in scCO_2_ [26,27,28,29], revealing that subtle differences in polymer polarity and molecular structure can significantly impact cloud point phenomena. Among the various available cosolvents, toluene has garnered significant attention due to its ability to enhance polymer solubility while remaining easily separable from the system upon depressurization [30,31,32,33]. Studies have demonstrated that incorporating toluene into scCO_2_ can substantially improve polymer dissolution by strengthening solute–solvent interactions [30,31,34]. However, most of these investigations focus on the density and phase behavior of scCO_2_–toluene mixtures or their critical loci, with limited systematic investigation of polymer cloud point behavior [30,35,36,37].

**Figure 1:**
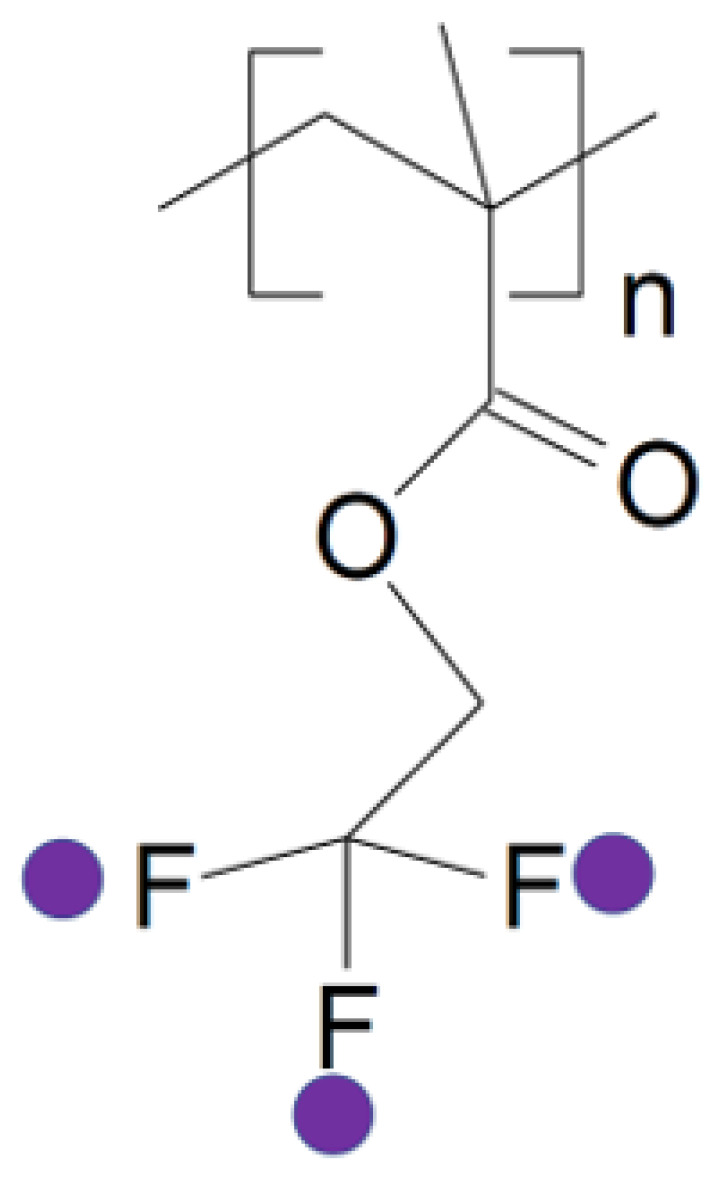
Chemical structure of poly(TFEMA). The polymer backbone originates from the methacrylate moiety, featuring an ester group linked to a 2,2,2-trifluoroethyl unit, which enhances its hydrophobicity and chemical resistance.

**Figure 2:**
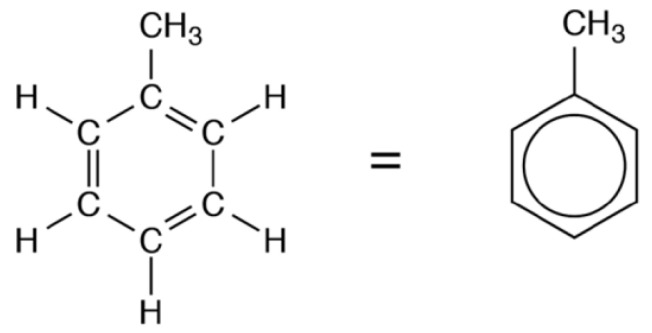
Chemical structure of toluene, an aromatic hydrocarbon often employed as a cosolvent in scCO_2_ due to its moderate polarity and π–π interaction capability, facilitating polymer dissolution.

Partially fluorinated polymers, such as poly(TFEMA), are known for their chemical inertness, hydrophobicity, and low surface energy [35,38,39,40]. As illustrated in Figure 1, poly(TFEMA) features a trifluoroethyl moiety in its side chain, which results in excellent moisture resistance and suitability for advanced coatings and membrane applications [38,39]. Although poly(TFEMA) dissolves in neat scCO_2_ at sufficiently high pressures [35], practical implementations often demand lower operating pressures and narrower temperature windows. Toluene (Figure 2), an aromatic cosolvent whose dispersion parameters closely match certain fluorinated segments, should effectively broaden the processing window by reducing cloud point pressures [31,32,34]. This approach aligns with recent efforts to optimize cosolvent composition for partially fluorinated materials in scCO_2_, as documented for various fluoropolymers [26,27].

Although partially fluorinated polymers such as poly(TFEMA) offer significant advantages for protective and antifouling coatings, as well as membrane and device applications [35,38], the phase behavior of these materials in scCO_2_ with aromatic cosolvents remains insufficiently characterized [41]. Kwon et al. [35] examined the high-pressure phase equilibria of CO_2_ + TFEMA and CO_2_ + poly(TFEMA); however, the role of toluene in further shifting the polymer’s cloud point has not been systematically investigated, particularly at moderate cosolvent fractions (e.g., up to 20 wt%).

Precise determination of polymer cloud points is essential for designing processes for a wide range of applications. Our group, for instance, utilized scCO_2_ to anneal and crystallize perovskite thin films for photovoltaic applications, achieving enhanced crystallinity at low temperature [42,43,44,45]. More recently, our group employed scCO_2_ to incorporate poly(TFEMA) into perovskite films to enhance environmental stability. scCO_2_-assisted crystallization of perovskite in the presence of a dissolved polymer resulted in polymer incorporation into the film rather than a separate coating [46]. In this context, the dissolution and solubility thresholds of fluoropolymers, such as poly(TFEMA), are critical for informing processing conditions.

This paper experimentally investigates the cloud point behavior of poly(TFEMA) in scCO_2_–toluene mixtures. Cloud point pressure measurements were systematically conducted over a temperature range of 31.5 °C to 50 °C, with cosolvent concentrations varying from 0 wt% to 20 wt%. Mixture densities were estimated using the AG–HGK equation of state for CO_2_ [47] and the Tait equation for toluene [48]. The results confirm that toluene serves as an effective cosolvent, significantly lowering cloud point pressures compared to neat scCO_2_ and thereby expanding the processing window for poly(TFEMA).

## 2. Results and Discussion

### 2.1. Cloud Point Pressures as a Function of Temperature

The cloud point pressures for poly(TFEMA) (3 wt%) in CO_2_–toluene mixtures were measured at temperatures ranging from 31.5 °C to 50 °C (304.65–323.15 K), with toluene concentrations of 0, 5, 10, 15, and 20 wt%. The experimental data are compiled in Table 1.

**Figure 3:**
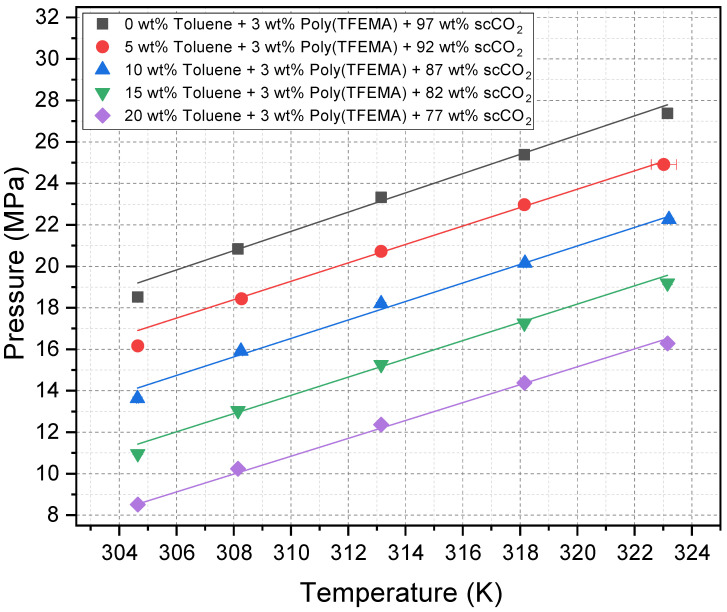
Cloud point pressure vs. temperature for 3 wt% poly(TFEMA) in scCO_2_ with varying toluene fractions (0–20 wt%). Error bars are within ±1% of reported values.

Figure 3 shows the corresponding cloud point pressure ($P_{\mathrm{cloud}}$) trends as a function of temperature for each toluene fraction. For all compositions, $P_{\mathrm{cloud}}$ increases linearly with temperature. This well-known behavior reflects the decreasing density of scCO_2_ (and its mixtures) at higher temperatures. Therefore, in order to maintain poly(TFEMA) solubility, higher pressures are required. At any fixed temperature, $P_{\mathrm{cloud}}$ decreases monotonically with increasing toluene concentration. Even at 5 wt%, toluene yields a substantial reduction in cloud point pressure relative to neat scCO_2_, reflecting the ability of toluene to enhance solvent–solute molecular interactions.

The polydispersity index (PDI) of the poly(TFEMA) used in this study is 2.1, with a number-averaged molecular weight of 31,283. The cloud point data depend on the molecular weight of the polymer, with higher-molecular-weight molecules exhibiting lower solubility. Therefore, the experimental cloud point curves shown in Figure 3 are influenced most strongly by the molecules at the higher end of the molecular weight distribution; these will drop out of solution first as the solvent pressure is reduced. The purpose of this paper is to illustrate the effectiveness of toluene as a cosolvent in reducing cloud point pressures, but it is important to recognize that the results presented here are molecular-weight dependent.

### 2.2. Effect of Toluene as a Cosolvent on Cloud Point Pressure

**Figure 4:**
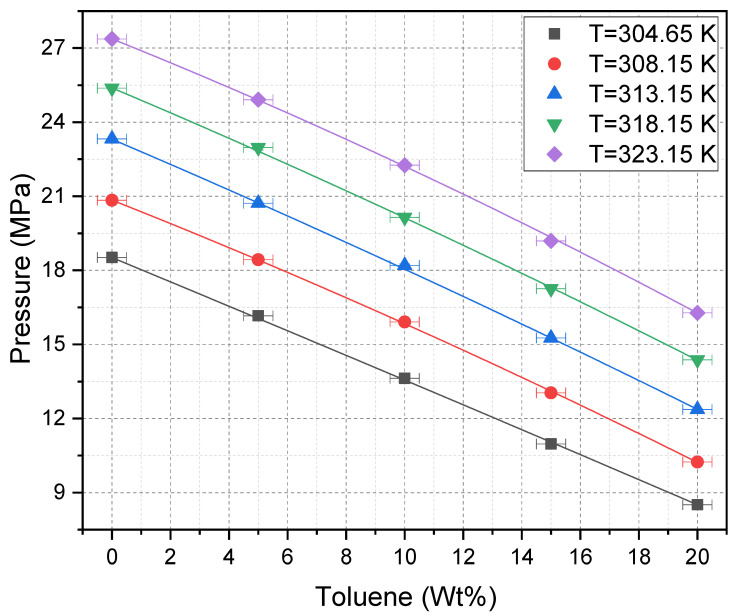
Cloud point pressure vs. toluene wt% at five isothermal conditions: 304.65, 308.15, 313.15, 318.15, and 323.15 K.

To further illustrate the effect of solvent composition, Figure 4 plots $P_{\mathrm{cloud}}$ against toluene weight percent at each isothermal condition of 31.5, 35, 40, 45, and 50 °C (304.65, 308.15, 313.15, 318.15, and 323.15 K, respectively). In each isotherm, $P_{\mathrm{cloud}}$ decreases nearly linearly with increasing toluene fraction. Although each isotherm is shifted to higher $P_{\mathrm{cloud}}$ values at higher temperatures, the overall trends remain consistent. Thus, cosolvent addition reliably compensates for the tendency of supercritical fluids to lose solvent power with increasing temperature. The horizontal error bars in the figure represent the estimated uncertainly in the toluene weight percent and, which is primarily due to the uncertainty in the weight of CO_2_ added to the view cell for the reasons described in Section 3.3.

Because carbon dioxide and toluene are both categorized as non-polar molecules, it is not immediately obvious why the addition of toluene leads to the observed increase in polymer solubility. Hansen solubility parameters (HSPs) are useful for comparing three types of molecular interactions: dispersion (van der Waals), polarity (dipole interactions), and hydrogen bonding. A comparison of the Hansen solubility parameters (HSPs) for carbon dioxide, toluene, and poly(TFEMA) shows that all three have very low propensity for dipole–dipole interactions and hydrogen bonding. However, toluene and poly(TFEMA) have nearly identical dispersion parameters—18 and 17.5, respectively—while the dispersion parameter for carbon dioxide is much lower at 15.6. For this reason, toluene is expected to have stronger molecular interactions with poly(TFEMA) and act as a more effective solvent.

### 2.3. Density Trends of scCO_2_–Toluene Mixtures at Cloud Point Conditions

**Figure 5:**
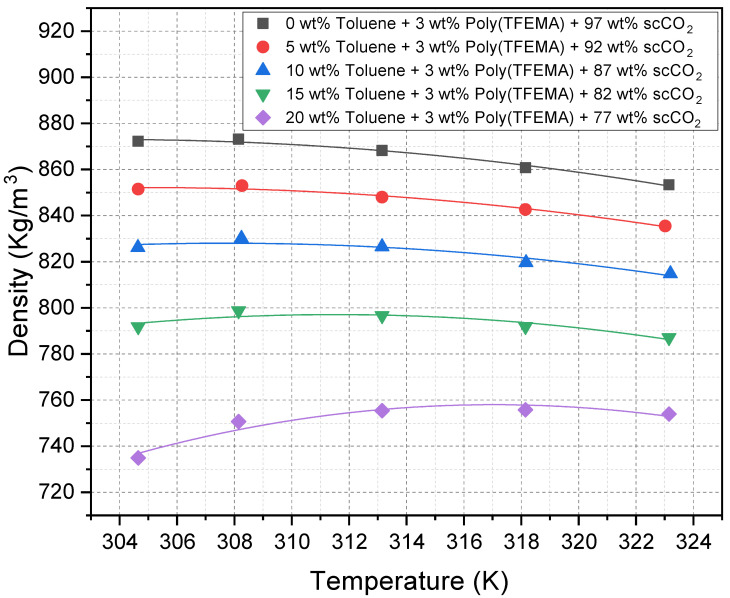
Calculated density of scCO_2_–toluene mixtures as a function of temperature at different toluene weight percentages (0–20 wt%).

Figure 5 presents the calculated densities of scCO_2_–toluene mixtures at various temperatures (304.65–323.15 K) and toluene concentrations (0–20 wt%). At lower concentrations of toluene, the solvent density at the cloud point decreases with increasing temperature, consistent with the expected thermal expansion of the solvents. However, at higher toluene concentrations, the solvent density at the cloud point exhibits more complex behavior, first increasing with temperature before leveling off and then decreasing at higher temperatures. This rather surprising non-monotonic behavior is likely due to the temperature dependence of the van der Waals interaction between toluene and poly(TFEMA). At lower temperatures, the van der Waals interaction is strongest, leading to polymer solubility at lower solvent density. However, as the temperature increases, the van der Waals interaction decreases, requiring a higher solvent density at the cloud point.

Another way of interpreting the data of Figure 5 is to observe the change in cloud point density at a fixed temperature. At the lowest temperature, there is a much larger change in cloud point density with increasing toluene concentration due to the stronger toluene–poly(TFEMA) van der Waals interactions. As the temperature is increased, the toluene impact on cloud point density decreases and the curves begin to converge. At any given temperature, the presence of toluene reduces the overall density needed for the poly(TFEMA) cloud point compared to neat scCO_2_.

Although density typically correlates with solvating power, the higher dispersion parameter of toluene compensates for the lower density. Hence, cloud point pressures are reduced (indicating better solvation) despite the mixture’s lower density. This underscores the complexity of polymer solubility in supercritical fluids, where both density and solvent polarity must be considered.

The observed reduction in cloud point pressure with increasing toluene concentration highlights the effectiveness of cosolvent addition in enhancing poly(TFEMA) solubility in scCO_2_–toluene mixtures. To ensure reproducibility and provide a rigorous basis for these findings, we now present the experimental methods, measurement protocols, and computational approaches used to obtain and analyze the reported data.

## 3. Materials and Methods

### 3.1. Materials

- Carbon Dioxide (CO_2_): High-purity (99.9995%) CO_2_ was purchased from Airgas (Philadelphia, PA, USA).
- Poly(Trifluoroethyl Methacrylate) [Poly(TFEMA)]: The fluorinated polymer was obtained from Specific Polymers (Castries, France). The polymer was used as received without further purification. This polymer exhibits a molecular weight (Mn) of 31,283 g/mol, a weight-average molecular weight (Mw) of 65,501 g/mol, and a polydispersity index (PDI) of 2.1, as specified by the supplier.
- Toluene: Toluene (99.8% purity) was purchased from Sigma Aldrich (St. Louis, MO, USA).

All chemicals were handled and stored according to manufacturer guidelines.

### 3.2. Experimental Setup

**Figure 6:**
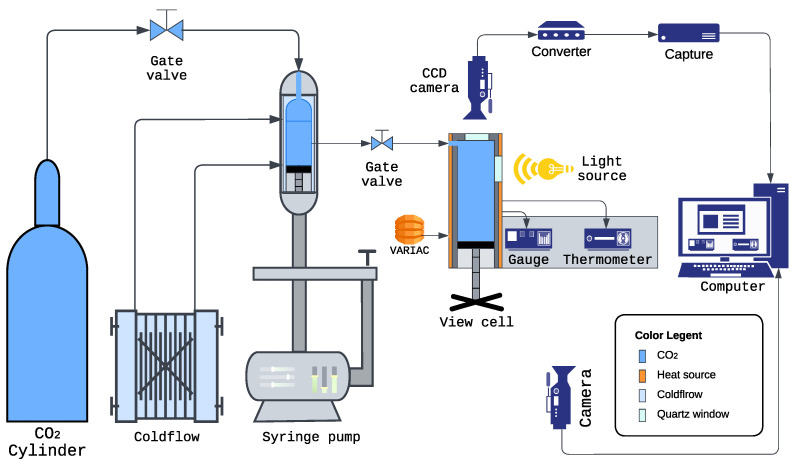
Schematic diagram of the experimental setup for cloud point measurements. The system integrates the CO_2_ supply, pressure and temperature control, and optical monitoring for accurate detection of cloud point phenomena.

Figure 6 is a schematic diagram illustrating the various components of the experimental apparatus used to obtain information on the phase behavior of poly(TFEMA) in a supercritical fluid solvent. The main component of the system is a variable-volume view cell with temperature and pressure control. The following sections provide a detailed description of each component and its role within the integrated system.

#### 3.2.1. Carbon Dioxide Delivery and Pressurization

High-purity CO_2_ was supplied from a carbon dioxide cylinder, which served as the primary source of pressurized fluid for the system. This cylinder was connected via high-pressure stainless steel tubing to a Teledyne Isco 260D syringe pump (Teledyne Isco, Lincoln, NE, USA). The syringe pump was used to pressurize the variable-volume view cell with CO_2_ to the desired operating levels. To maximize CO_2_ loading efficiency within the pump, a NESLAB CFT-25 refrigerated recirculator (Neslab Instruments, Inc., Portsmouth, NH, USA) was employed to reduce the reservoir temperature and increase the CO_2_ density, allowing larger quantities of CO_2_ to be stored in the syringe pump with fewer reload cycles. This arrangement provided a stable and continuous supply of pressurized CO_2_ for the subsequent experimental stages.

#### 3.2.2. Phase Monitor (Variable-Volume View Cell)

Pressurized CO_2_ from the syringe pump was directed into the SFT Phase Monitor (Supercritical Fluid Technologies, Inc., Newark, DE, USA); a high-pressure view cell with a 30 mL maximum internal volume, sealed using an isolation valve. This variable-volume cell was used to control the pressure by adjusting its internal piston position, which advances at a rate of 14 turns per inch, displacing 0.36 mL per turn.

Temperature regulation was achieved through a proportional–integral–derivative (PID) controller attached to a resistance temperature detector (RTD), providing a temperature sensing accuracy of ±0.5% °C and ensuring stable experimental conditions. The quartz windows, measuring 1/2 inch thick and 7/8 inch in diameter, allow safe operation up to 10,000 psi and 300 °C, enabling reliable high-pressure and high-temperature phase behavior studies.

The Phase Monitor enabled direct visual observation of the polymer solution, making it possible to identify cloud point conditions by detecting the onset of turbidity, indicative of a transition from a single-phase to a multi-phase system.

#### 3.2.3. Temperature Control

The Phase Monitor’s temperature was regulated using a combination of a heavy-insulated industrial heating tape (Model AWD-051-060) from HTS/Amptek (Stafford, TX, USA, 120V, 312W) and a Powerstat 3PN117C variable autotransformer (Warner Electric, South Beloit, IL, USA, 120V, 12A) (Variac). The heating tape was wrapped around the cylindrical section of the cell, while the Variac controlled the power supplied to the tape, enabling precise adjustment of the cell temperature between 31.2 °C and 50 °C. The temperature was continuously monitored using a thermocouple, ensuring that the experimental conditions remained within the specified range. This dual heating control mechanism provided thermal stability, crucial for reproducible measurements.

#### 3.2.4. Pressure Measurement

Real-time pressure monitoring was achieved using an OMEGA PX309-10KGI high-performance pressure transducer (Omega Engineering Inc., Norwalk, CT, USA), rated for the experimental pressure range. The transducer output was displayed on an OMEGA DP400TP high-speed panel meter (Omega Engineering Inc., Norwalk, CT, USA), offering a system accuracy of ±0.25% of full scale, considering the transducer’s combined linearity, hysteresis, and repeatability. This setup enabled accurate measurement and real-time visualization of internal pressure conditions within the Phase Monitor, ensuring precise control of the experiment.

#### 3.2.5. Optical Imaging and Video Recording

The cloud point was identified by observing visual changes in the solution’s turbidity. For this purpose, two cameras were utilized:

- Vanxse CCTV Mini HD 1/3 CCD 960H Auto Iris Camera (Model BX2812, Shenzhen Kaixing Security Technology Co., Ltd., Shenzhen, China, NTSC): This camera was positioned to capture the optical appearance of the solution through the quartz window of the Phase Monitor. Changes in clarity, such as the onset of turbidity, indicated the cloud point.
- Angetube 1080P Webcam (Model XZC827, Angetube, Shenzhen, China, USB): This camera recorded the digital displays of the DP400TP panel meter (showing real-time pressure in psi) and the thermometer (showing temperature in °C).

Both camera feeds were synchronized using OBS Studio 30.0.2 (OBS Project, Online Open-Source Community), which combined them into a single video file. This allowed simultaneous recording of the cloud point event and its corresponding temperature and pressure data, which provided a comprehensive record for analysis.

#### 3.2.6. Data Acquisition and Analysis

The computer served as the central hub for data acquisition, where the synchronized video output from both cameras was stored. The OBS Studio software enabled real-time visualization and recording of the three critical elements:

1. The visual appearance of the polymer solution within the Phase Monitor.
2. The pressure reading displayed on the DP400TP panel meter.
3. The temperature reading from the system thermometer.

This unified recording approach ensured that the experimentalist could observe and analyze all relevant parameters simultaneously, simplifying the post-experiment analysis and interpretation of the results.

#### 3.2.7. Overall System Integration

Figure 6 illustrates the interconnected components of the experimental setup. CO_2_ was delivered from the cylinder to the chilled syringe pump and then transferred to the Phase Monitor. The temperature of the Phase Monitor was regulated by the heating system, while pressure measurements were taken using the OMEGA transducer and panel meter. Visual observations were captured by the CCD camera, and operational data (pressure and temperature) were recorded by the webcam. This integrated system ensured high-precision cloud point measurements, facilitating detailed analysis of the fluoropolymer phase behavior in supercritical solvents.

### 3.3. Experimental Procedure

The experiments were carried out at a fixed polymer concentration of 3 wt% and varying toluene cosolvent concentrations (0 wt%, 5 wt%, 10 wt%, 15 wt%, and 20 wt%). The cloud point was determined over a temperature range from 31.5 °C (304.65 K) to 50 °C (323.15 K). Particular care was taken to ensure consistent initial conditions inside the view cell of the Phase Monitor and to accurately determine the mass of scCO_2_. The resulting data enabled a systematic investigation of the effect of toluene as a cosolvent on the solubility and phase behavior of poly(TFEMA) in scCO_2_.

#### 3.3.1. Reference State and CO_2_ Loading

All experiments started from a reference state of 31.2 °C (304.35 K) and 1100 psi (7.58 MPa) in a nominal 30 mL Phase Monitor cell. Under these conditions, the density of CO_2_ was calculated to be approximately 623.24 kg·m^−3^ (as determined from an equation-of-state calculator), giving a mass of about 18.723 g of CO_2_ [47]. We adopted this mass (18.723 g) as the basis for calculating the solvent composition (scCO_2_ plus toluene) and the polymer mass necessary to achieve (3 wt%).

To independently estimate the CO_2_ loading experimentally, we employed a Parr Instrument Company pressure vessel (Model 4768, Parr Instrument Company, Moline, IL, USA). First, the vessel was charged with CO_2_ and weighed using an American Weigh Scales KGX-10 high-capacity precision scale (American Weigh Scales, Inc., Cumming, GA, USA). Then, a portion of the CO_2_ was transferred into an intermediary pipe, weighed again, and finally introduced into the Phase Monitor cell. By recording the mass loss from the Parr vessel after transferring CO_2_ to the Phase Monitor and ensuring that the Phase Monitor cell reached the set reference conditions (31.2 °C, 1100 psi, 30 mL volume), we obtained a total scCO_2_ mass of 19.8 g. This value is 1.08 g higher than the value obtained from the equation-of-state density calculation and was used to inform our uncertainty analysis and error bars.

#### 3.3.2. Compositions and Preparation

Five cosolvent formulations were studied, each containing approximately 3 wt% poly(TFEMA) and 0–20 wt% toluene. The composition balance was scCO_2_. The five weight percentages studied were as follows:

(1) 0 wt% toluene + 3 wt% Poly(TFEMA) + 97 wt% CO_2_
(2) 5 wt% toluene + 3 wt% Poly(TFEMA) + 92 wt% CO_2_
(3) 10 wt% toluene + 3 wt% Poly(TFEMA) + 87 wt% CO_2_
(4) 15 wt% toluene + 3 wt% Poly(TFEMA) + 82 wt% CO_2_
(5) 20 wt% toluene + 3 wt% Poly(TFEMA) + 77 wt% CO_2_

While the mass of the polymer and toluene cosolvent were accurately determined by weighing each before deposition into the view cell, the mass of the scCO_2_ could only be estimated from the delivery pressure and temperature. For these experiments, we assumed that the mass of scCO_2_ remained constant at 18.723 g (based on the calculated density at 31.2 °C and 1100 psi and a cell volume of 30 mL). That is, we assumed that the presence of the polymer and cosolvent in the bottom of the view-cell chamber had a small influence on the scCO_2_ mass transferred at the reference pressure and temperature. At 1100 psi, the cosolvent and polymer are not completely dissolved in the scCO_2_ and should, therefore, not contribute significantly to the view-cell total pressure. However, they do occupy a small fraction of the view cell’s internal volume, which was difficult to estimate in the presence of scCO_2_ due to partial dissolution. We also attempted to determine the mass of scCO_2_ transferred into the view cell by weighing an intermediary transfer vessel before and after pressurization, but the low pressure differential and longer time required to stabilize the temperature resulted in cosolvent and polymer backflow into the transfer vessel. For this reason, we assumed a fixed mass of scCO_2_ and estimated the error to be within 5%. From the fixed mass of scCO_2_, the required masses of polymer and toluene were calculated to preserve a 3 wt% polymer fraction and the desired wt% of toluene. The specific compositions used in the experiments were as follows:

- 0 wt% Toluene: 0.579 g of poly(TFEMA) + 18.723 g of scCO_2_.
- 5 wt% Toluene: 1.01753 g (1.18 mL) of toluene + 0.61052 g of poly(TFEMA) + 18.723 g of scCO_2_.
- 10 wt% Toluene: 2.15202 g (2.4957 mL) of toluene + 0.64561 g of poly(TFEMA) + 18.723 g of scCO_2_.
- 15 wt% Toluene: 3.42486 g (3.9718 mL) of toluene + 0.68497 g of poly(TFEMA) + 18.723 g of scCO_2_.
- 20 wt% Toluene: 4.86301 g (5.6396 mL) of toluene + 0.72945 g of poly(TFEMA) + 18.723 g of scCO_2_.

All experiments began by placing the appropriate amount of poly(TFEMA) and (when required) toluene into the Phase Monitor cell:

(a) No toluene (0 wt%): A mass of 0.579 g of poly(TFEMA) alone was weighed on a Fisher Scientific (Waltham, MA, USA) Accu-124 analytical balance, then introduced into the cell.
(b) Toluene-containing mixtures (5–20 wt%): The predetermined mass of poly(TFEMA) was first weighed and added to the cell. Afterward, the measured toluene (e.g., 1.18 mL for 5 wt%) was carefully pipetted into the cell.
(c) Sealing and purging: After loading the cell with polymer and toluene (when applicable), the cell was sealed. CO_2_ was introduced gradually (at a flow rate of 10 mL/min) until the internal pressure reached roughly 600–700 psi (4.14–4.83 MPa). A brief purge was performed through the outlet valve to remove air; this was performed carefully so as to avoid loss of toluene or polymer. The outlet valve was then closed.
(d) Pressurizing to reference state: The cell was wrapped with flexible heating tape and maintained at 31.2 °C using a Variac. The syringe pump was then used to increase the cell pressure to 1100 psi at a controlled flow (10–20 mL/min). Once the reference state (31.2 °C, 1100 psi) was stable, the inlet valve was closed to isolate the system.

#### 3.3.3. Cloud Point Determination

Cloud points were measured by adjusting the view-cell volume and cell pressure while visually monitoring the solution turbidity along isotherms at 31.5, 35, 40, 45, and 50 °C.

**Figure 7:**
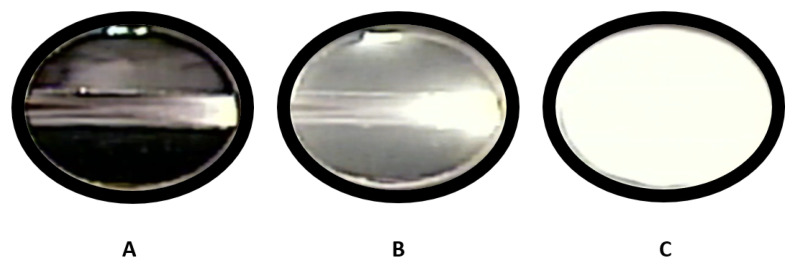
Camera images through the quartz window of the Phase Monitor showing (**A**) a fully dissolved (single-phase) polymer solution, (**B**) the onset of cloud formation (cloud point), and (**C**) significant phase separation (polymer precipitation).

Figure 7 shows the visual sequence for the polymer + scCO_2_ + toluene system observed through the cell’s quartz window. For each temperature and composition, the following procedure was used:

(1) Single-phase formation: The internal volume of the cell was decreased (i.e., the piston moved inward) to increase pressure until the mixture appeared optically transparent in the cell’s quartz window. This corresponded to a single-phase region, as illustrated in Figure 7A.
(2) Expansion to turbidity (cloud point): Next, the piston was slowly retracted, reducing the pressure at a controlled rate, until the first discernible turbidity was observed in the window (onset of clouding). This pressure was noted as $P_{\mathrm{cloud}}$. The visual appearance at this point is illustrated in Figure 7B, where the polymer-rich phase begins to form but the solution is only partially cloudy.
(3) Further expansion: If the volume was expanded further, the system entered a fully two-phase region, exhibiting a milky or opaque appearance, as shown in Figure 7C. At this stage, the polymer is largely precipitated from the fluid phase.
(4) Repetitions: Each temperature–composition combination was measured at least six times to ensure reproducibility. The mean $P_{\mathrm{cloud}}$ value is reported, with typical standard deviations around ±1%.

The methodology described above was carried out uniformly for all five toluene compositions (0–20 wt%), ensuring that changes in polymer solubility could be attributed solely to the variation in the percentage of cosolvent weight.

### 3.4. Density Calculations

The density of the binary solvent (scCO_2_ + toluene) was calculated over the range of temperatures and pressures used in the cloud point study to better understand the influence of solvent chemical composition versus solvent density on the observed change in polymer solubility.

#### 3.4.1. CO_2_ Density: AG–HGK Equation of State

CO_2_ densities were evaluated using the Altuin–Gadetskii (AG) far-field equation, combined with the Haar–Gallagher–Kell (HGK) approach near the critical region, collectively referred to here as the AG–HGK equation of state [47]. This hybrid equation of state (EoS) is particularly suited for describing CO_2_ in or near the critical region, where non-ideal behavior is pronounced. By capturing both far-field (AG) and near-critical (HGK) corrections, the AG–HGK model yields reliable density estimates over the relevant range of temperatures (31.5–50 °C) and pressures (8–30 MPa) of our study.

#### 3.4.2. Toluene Density: Tait Equation

Toluene densities were computed via the Tait equation, which provides a flexible semi-empirical description of liquid compressibility across a wide range of temperatures and pressures [48,49]. The general form is

(1) $$\rho \left(P,T\right)\;=\;\frac{\rho_{0}\left(T\right)}{1-C\log_{10}\;\left(\frac{P+B(T)}{P_{0}+B\left(T\right)}\right)},$$

where $P_{0}=0.1$ MPa, C=0.2219, and B(T) is a temperature-dependent term encapsulating the compressibility of toluene. The reference density $\rho_{0}\left(T\right)$ and B(T) are both expressed as quadratic polynomials in *T*:

(2) $$\rho_{0}\left(T\right)=\sum_{i=0}^{2}a_{i}T^{i}=a_{0}+a_{1}T+a_{2}T^{2}.$$

(3) $$B\left(T\right)=\sum_{i=0}^{2}b_{i}T^{i}=b_{0}+b_{1}T+b_{2}T^{2}.$$

The coefficients $a_{i}$ and $b_{i}$ are listed in Table 2 [48].

In expanded form, the density of toluene at any (P,T) can thus be written as

(4) $$\rho_{\mathrm{Tol}}\left(P,T\right)\;=\;\frac{\sum_{i=0}^{2}a_{i}T^{i}}{1\;-\;C\log_{10}\;\left(\frac{P+\sum_{i=0}^{2}b_{i}T^{i}}{P_{0}+\sum_{i=0}^{2}b_{i}T^{i}}\right)}.$$

The above formulation reproduces our density data for toluene across the range of conditions relevant to the cloud point measurements [48].

#### 3.4.3. Mixture Density: Mass-Weighted Mixing Law

After computing individual densities for CO_2_ and toluene at the required *P*–*T* conditions, we estimate the density of the binary solvent (scCO_2_ + toluene) using the following mass-weighted mixing rule:

(5) $$\rho_{\mathrm{mix}}=\sum_{i}x_{i}\cdot \rho_{i}=x_{CO_{2}}\cdot \rho_{CO_{2}}+x_{Tol}\cdot \rho_{Tol},$$

where $x_{CO_{2}}$ and $x_{Tol}$ are the respective mass fractions of CO_2_ and toluene, while $\rho_{{\mathrm{CO}}_{2}}$ and $\rho_{\mathrm{Tol}}$ are the densities predicted by the AG–HGK EoS and the Tait equation, respectively. This mixing law, although approximate, provides a reasonable first-order estimate of the overall solvent density in our system.

## 4. Conclusions

In this study, we investigated the cloud point behavior of a 3 wt% solution of poly(TFEMA) (PDI = 2.1) in scCO_2_–toluene mixtures over 31.5–50 °C, with toluene concentrations ranging from 0 to 20 wt%. Given the broad molecular weight distribution, higher-molecular-weight fractions likely governed the observed cloud point transitions as they precipitated first.

Key findings include the following:

- Cloud point trends: Cloud point pressures rise linearly with temperature, but fall with increasing toluene fraction, demonstrating how cosolvent addition effectively reduces the pressure required for polymer dissolution.
- Density: Toluene lowers the overall mixture density but boosts solvent–solute interactions, primarily due to the similarity in dispersion parameters between toluene and poly(TFEMA), leading to enhanced solvating capability for fluoropolymers such as poly(TFEMA).
- Process implications: At the highest cosolvent fraction of 20 wt%, we observed up to an approximately 40% reduction in cloud point pressure compared to neat scCO_2_, demonstrating a significant expansion of the processing window. These insights form a basis for designing supercritical fluid processes with lower pressure requirements, translating to potential energy savings and broader industrial applicability.

In conclusion, careful selection of the toluene content in scCO_2_ enables fine control of polymer solubility, striking a balance between density and molecular interaction effects. The methodologies described—AG–HGK for CO_2_, Tait equation for toluene, and a mass-weighted mixing rule—provide a comprehensive and practical framework for predicting mixture densities, thereby facilitating deeper understanding of the cloud point phenomena observed in supercritical fluid operations.

## Figures and Tables

**Table 1:** Cloud point pressures for 3 wt% poly(TFEMA) in scCO_2_–toluene at different temperatures. Each value is an average of at least six measurements (±1%).

Temp.	Cloud Point Pressure (MPa)				
**(°C)**	**0 wt% Tol**	**5 wt% Tol**	**10 wt% Tol**	**15 wt% Tol**	**20 wt% Tol**
31.5	18.52	16.16	13.63	10.97	8.51
35.0	20.84	18.43	15.91	13.04	10.24
40.0	23.32	20.72	18.20	15.26	12.37
45.0	25.38	22.97	20.15	17.26	14.38
50.0	27.37	24.91	22.26	19.19	16.28

**Table 2:** Parameters for Equations (2) and (3) to calculate toluene densities with the Tait equation (Equation (1)) from 296 to 535 K and 10 to 300 MPa.

**Equation (4)**: *ρ*_0_(*T*)/kg·m^−3^	
$10^{-3}\cdot a_{0}$/kg·m^−3^	1.0267
$10\cdot a_{1}$/kg·m^−3^·$K^{-1}$	−2.5956
$10^{3}\cdot a_{2}$/kg·m^−3^·$K^{-2}$	−1.0127
**Equation (5)**: B(T)/MPa	
$10^{-2}\cdot b_{0}$/MPa	3.9435
$b_{1}$/MPa·K^−1^	−1.3143
$10^{3}\cdot b_{2}$/MPa·K^−2^	1.1164

## Data Availability

Data supporting the findings of this study are available from the corresponding author upon reasonable request.

## Abbreviations

AG	Altuin–Gadetskii
AG–HGK	Altuin–Gadetskii–Haar–Gallagher–Kell
CO_2_	Carbon dioxide
HSPs	Hansen solubility parameters
HGK	Haar–Gallagher–Kell
PDI	Polydispersity index
scCO_2_	Supercritical carbon dioxide
TFEMA	Trifluoroethyl methacrylate
Tol	Toluene
wt%	Weight percent
K	Kelvin
kg·m^−3^	Kilograms per cubic meter (density unit)
MPa	Megapascal
psi	Pounds per square inch
T	Temperature
B(T)	Tait equation parameter dependent on temperature
C	Compressibility parameter in Tait equation
P_0_	Reference pressure (0.1 MPa)
P_cloud_	Cloud point pressure
ρ	Density
$\rho_{0}\left(T\right)$	Reference density at temperature *T*
CCD	Charge-coupled device
HD	High definition
PID	Proportional–integral–derivative (controller)
RTD	Resistance temperature detector
EoS	Equation of state
OBS	Open Broadcaster Software
